# Combining Transcranial Direct Current Stimulation with Exercise to Improve Mobility, Stability, and Tremor Management in 25 Individuals with Parkinson’s Disease

**DOI:** 10.3390/neurolint16060093

**Published:** 2024-10-28

**Authors:** Fabrício D. de Almeida, Yiyu Wang, Rodrigo C. de Mello Pedreiro, Ana Carolina B. Brizzi, Shirley F. Campos, Melina P. Sales, Deanna M. Kennedy, Osmar Pinto Neto

**Affiliations:** 1 Department of Biomedical Engineering, Anhembi Morumbi University, São José dos Campos 12247-016, SP, Brazil; fabrisduarte@hotmail.com (F.D.d.A.); carol.brisola.cb@gmail.com (A.C.B.B.); shirleycampos30@hotmail.com (S.F.C.); melina.sales@unitau.br (M.P.S.); 2 Department of Anatomy, Federal Rural University of Rio de Janeiro, Seropédica 23890-000, RJ, Brazil; 3 Department of Psychology, Princeton University, Princeton, NJ 08540, USA; yw4722@princeton.edu; 4 Departament of Physical Education, Estácio de Sá University, Teresópolis 25963-150, RJ, Brazil; rodrigocmp1@gmail.com; 5 Arena235 Research Lab, São José dos Campos 12246-876, SP, Brazil; 6 Departments of Psychology and Physical Therapy, Universidade de Taubaté (Unitau), Taubaté 12020-040, SP, Brazil; 7 Department of Kinesiology and Sport Management, Texas A&M University, College Station, TX 77845, USA; hpedmk@tamu.edu; 8 Department of Kinesiology, California State University San Marcos (CSUSM), San Marcos, CA 92096, USA; 9 Center of Innovation Technology and Education-CITÉ, São José dos Campos 12247-016, SP, Brazil

**Keywords:** neuromodulation, non-invasive, physical activity

## Abstract

Background/Objectives: Parkinson’s disease (PD) is a neurodegenerative disorder characterized by tremors, balance impairments, and mobility limitations. Innovative approaches like combining transcranial direct current stimulation (tDCS) with exercise show promise in addressing these symptoms. This study investigates the effects of exercise combined with tDCS on mobility and tremor management in PD patients. Methods: Twenty-five individuals aged 60−75 (66.6 ± 7.33), diagnosed with PD (Hoehn and Yahr stage 2−3), were assigned to three groups in a randomized controlled design: exercise with active tDCS (*n* = 8), exercise with sham tDCS (*n* = 8), and a control group (*n* = 9). Dual-task training sessions focusing on walking speed, balance, and force control were conducted over ten sessions. Results: No significant differences were detected across the groups for grip strength or force control measures (*p* > 0.05). Significant improvements were observed in the intervention group: the Timed Up and Go (TUG) test showed a significant reduction in time (mean difference = 2.498 s, *p* < 0.001, ηp^2^ = 0.331); anterior–posterior displacement significantly increased (mean difference = 21.375 mm, *p* = 0.0269, ηp^2^ = 0.303); and force-tremor decoupling improved, with coherence in the 1−4 Hz band significantly decreasing (*p* = 0.0067). Finally, changes in TUG from post- to pre-treatment values were significantly positively correlated with the changes in coherence (R = 0.468, *p* = 0.018). Conclusions: Combining tDCS with exercise enhances mobility and tremor management in PD patients. These findings support the potential for such interventions to improve functional outcomes and quality of life for individuals with PD.

## 1. Introduction

Parkinson’s disease (PD) is a progressive neurodegenerative disorder that significantly impacts motor function and is characterized by tremors, balance impairments, and mobility limitations. In addition, cognitive impairment is also common in PD patients. Cognitive deficits can range from mild cognitive impairment (PD-MCI) to more severe forms, including Parkinson’s disease dementia (PDD), which affects approximately 30% of individuals with PD over time [1]. These cognitive changes can significantly impact executive functions, memory, and decision-making abilities, further complicating daily activities and overall quality of life [2].

These symptoms pose considerable challenges to daily activities and overall quality of life. Traditional treatments often fall short in addressing the complex nature of the disease, necessitating innovative strategies to improve patient outcomes [3,4,5,6]. Combining transcranial direct current stimulation (tDCS) with exercise has emerged as a promising approach to address these complex symptoms [7,8,9]. tDCS is known for its ability to modulate cortical excitability [10,11], potentially enhancing the effects of exercise-induced neuroplasticity [12,13]. Exercise alone has well-documented benefits for individuals with PD, including slowing disease progression and improving quality of life [14,15]. For example, exercise can enhance balance stability, as evidenced by reduced medial–lateral and anterior–posterior displacement during static tasks, leading to fewer falls and greater confidence in performing daily activities [16].

Improvement in mobility is another critical benefit of exercise, often measured by the Timed Up and Go (TUG) test [17,18]. This test evaluates the time it takes for an individual to rise from a chair, walk a short distance, turn, return, and sit down again. Better TUG performance indicates enhanced mobility and agility, enabling patients to move more efficiently and independently [19].

Tremors, a hallmark motor symptom of PD, significantly impact daily activities and motor control. As tremor severity increases, patients experience reduced motor control [20,21]. Tremor–force decoupling, the ability to generate force independently of tremor activity, is crucial for maintaining motor control and executing precise movements [22,23,24]. Improvements in tremor–force decoupling suggest better management of tremor symptoms, resulting in smoother and more coordinated movements [25].

These benefits collectively improve functional independence and quality of life for individuals with PD. Augmenting exercise with tDCS can enhance these benefits further, promoting sustained improvements in motor function and mobility [26]. Transcranial direct current stimulation (tDCS) has been increasingly explored as a non-invasive intervention for improving both motor and cognitive symptoms in Parkinson’s disease. Numerous studies have demonstrated that tDCS can enhance motor performance, such as improving upper limb function, reducing motor symptoms measured by the Unified Parkinson’s Disease Rating Scale (UPDRS), and modulating neural activity in motor-related areas [27]. Despite promising findings, there remains significant variability in tDCS parameters, including electrode placement, stimulation intensity, and frequency, which complicates the understanding of its long-term effects and the mechanisms behind these improvements. This study builds on the existing literature by combining tDCS with an exercise protocol and focusing specifically on tremor–force decoupling and motor control improvements in PD. Both tDCS and exercise are non-invasive interventions with minimal side effects when administered correctly, making this combined approach particularly appealing for patients who may be reluctant to undergo invasive procedures or have contraindications for medications such as Levodopa.

Furthermore, understanding the underlying mechanisms associated with the combined interventions of tDCS and exercise can provide valuable insights into optimizing treatment protocols for PD. This study aims to determine the effects of combining tDCS with exercise on functional mobility and tremor control, exploring the potential for significant improvements in motor function and overall well-being for individuals with PD.

## 2. Materials and Methods

### 2.1. Participants

Twenty-five individuals diagnosed with PD, aged 60 to 75 years (mean age = 66.6 ± 7.33 years, 20 females and 16 males), participated in the study. All participants were between stages 2 and 3 on the Hoehn and Yahr scale, indicating moderate disease severity [28]. The study adhered to the Guidelines and Regulatory Standards for Research Involving Human Subjects, issued by the National Health Council, Ministry of Health, Brazil, in October 1996. Ethical approval was granted by the Ethics Committee of Anhembi Morumbi University, São Paulo, Brazil, under approval number 3,903,038. The participants included in our study were all previously diagnosed with Parkinson’s Disease by their respective specialty physicians.

The study followed a randomized controlled design. Participants were randomly assigned to one of three groups (intervention, sham, or control). The randomization sequence was generated without knowledge of the participants’ testing order to ensure allocation concealment. The study utilized simple random sampling for participant allocation, and the participants were unaware of their assigned groups. Also, it was single-blinded, as the researchers manually turned off the electrical stimulation in the Sham condition. Inclusion criteria included participants who (a) have a diagnosis of Parkinson’s disease (PD), (b) are aged between 60 and 75 years, (c) are at Hoehn and Yahr stages 2 or 3, (d) have levels of comprehension and cooperation compatible with performing the proposed activities, (e) do not have other associated pathologies, (f) are in the “On” phase of medication, and (g) agree to participate in the study by signing the informed consent form (ICF). Participants’ comprehension and cooperation were assessed subjectively during the initial anamneses, and they were given a brief trial to ensure they understood the experimental protocol and task demands. Exclusion criteria included individuals with (a) orthopedic deformities, (b) other neurological diseases, (c) metal in the head, surgical clips, or metal plates, (d) implanted devices such as pacemakers, Deep Brain Stimulation (DBS), or cochlear implants, and (e) the need for walking aids.

Anthropometric data (weight and height) were measured for all participants. All participants demonstrated sufficient comprehension of the tasks and were in the “ON” phase of their medication, taking Prolopa 200/50 and Levodopa. Table 1 shows all participants’ medication and the Levodopa Equivalent Daily Dose (LEDD).

Evaluations were conducted as follows: On the first day, patients were registered, and anthropometric data were collected. On the subsequent day, force control, tremor assessment, stabilometric assessment, and the TUG test were performed. Over the next two weeks, subjects in the intervention and sham groups participated in 10 sessions of a specific exercise protocol. Only the intervention group received tDCS in conjunction with the training, while the sham group underwent the same activity with tDCS turned off. The control group did not participate in any exercise sessions; they were asked to continue their usual daily routines and return after two weeks. All participants underwent a second round of evaluations two weeks after the initial assessments, including force control, tremor assessment, stabilometric assessment, and the TUG test.

### 2.2. Sample Size Computation

Sample size calculations were performed using G*Power 3.1.9.7 software, requiring a minimum of 24 people for our research. For this calculation, we considered F tests for the test family, analysis of variance (ANOVA) with repeated measures, and within-between interaction for three groups (intervention, sham, control), two time periods (pre and post), a power of 0.80, correlation among repeated measures of 0.5, and an effect size f of 0.35 [29,30]. We recruited 25 participants.

### 2.3. Force Control and Acceleration

A secondary cognitive task can increase resting tremors in PD patients [31]. Thus, to quantify force tremor coupling, we examined force control on one hand while quantifying tremor on the other hand. Participants were required to sustain the palm grip position for 20 s, keeping a constant force of 20% of the maximum voluntary contraction (MVC) [32,33]. Accelerometers were placed in the most-affected hand, while the less-affected hand performed the constant force task. We collected grip force and acceleration data from the contralateral hand resting in a natural posture.

### 2.4. Hand Grip Strength

Force data were collected using a manual grip dynamometer (Vernier Software & Technology, Beaverton, OR, USA). Subjects stood before a 14-inch LCD monitor displaying a graph and held the dynamometer with their dominant hand. They exerted maximum force with one hand, generating force in finger flexion (palmar grip) for 3 s. Three force spikes were performed for each hand, and the average was calculated. MVC was quantified as the average force over a minimum of one second of the highest attempt. For the constant force control test, subjects sustained the palm grip position for 20 s at 20% of MVC [32,33].

### 2.5. Force Data Analysis

Force signals were band-pass filtered between 0.05 Hz and 10 Hz (Butterworth, order 4) and detrended using Matlab 7.0.1 (MathWorks Inc., Natick, MA, USA). Force variability was estimated as the standard deviation (SD of force) and coefficient of variation (CV of force) of the detrended force in the time domain, and force error as the root mean square error (RMSE).

### 2.6. Accelerometry

A triaxial accelerometer (model 3D-BTA, Vernier) with a range of ±49 m/s^2^ (±5 g) and frequency response of 0−100 Hz was placed on the contralateral hand using elastic bandages. Resultant acceleration signals were band-pass filtered between 0.05 Hz and 10 Hz and detrended in Matlab 7.0.1. Auto-spectral analysis was performed using Welch’s averaged periodogram method. The window size (256) was selected to approximate a 0.3906 Hz frequency bin. Tremor modal frequency was estimated between 4−16 Hz, as higher frequencies above 4 Hz relate to physiological and pathological tremors [34,35,36]. Approximate entropy (ApEn) was calculated on the force signal using parameter settings: m = 2; r = 0.2 × standard deviation of the signal [36,37,38].

### 2.7. Force and Acceleration Coherence

The coherence between grip force and contralateral acceleration signals was estimated to examine the global impact of tremors in force control. We employed the Welch technique to compute their power spectra and cross-spectrum. The method begins by segmenting the time series into overlapping windows of size windSize, where each segment is tapered using a specifically designed window function, known as the Matviyenko taper, to reduce spectral leakage [39]. The discrete Fourier transform (DFT) is computed for each tapered segment, and the individual periodograms are subsequently averaged, enhancing the statistical reliability of the spectral estimates. The result is refined by convolving with a smoothing kernel to obtain power spectral densities pXX and pYY for force and acceleration, respectively, and their cross-spectrum pXY. The estimated spectra are limited to a predefined frequency range of up to 20 Hz. The spectral analysis was crucial for our subsequent coherence calculation, where the magnitude-squared coherence provides insights into the frequency–domain correlation between the two signals. Coherences were estimated for the frequency bands 0–1, 1–4, 4–8, and 8–12 Hz.

### 2.8. Functional Limit Test

A fixed force platform, S-PLATE (Medicapteurs, France), was used with dimensions of 610 mm in width and 580 mm in depth, and an active area of 400 × 400 mm featuring 1600 resistive sensors. Each sensor measured 0.64 cm^2^, allowing for stabilometric analysis by recording center-of-pressure oscillations. Data were collected at 100 Hz, and participants were positioned orthostatically on the platform, barefoot, and focusing their gaze on a fixed point. Patients were instructed to lean their trunks forward for 10 s until reaching their functional limit, followed by 10 s of leaning backward to their functional limit. Anterior–posterior (AP) positional data were band-pass filtered between 0.005 Hz and 5 Hz (Butterworth, order 4) in Matlab 7.0.1 (MathWorks Inc.). The processed data estimated the maximum amplitude of anterior–posterior center-of-pressure displacement (AP Displacement) [40].

### 2.9. Timed up and Go (TUG)

The TUG is a widely recognized tool for assessing functional mobility and evaluating balance and gait. Additionally, it has been identified as a significant predictor of fall risk [41]. The participant begins the test seated in a standard armchair with a seat height of approximately 46 cm and armrests. Their back should be against the chair, feet flat on the floor, and arms resting on the armrests. On the command “Go”, the participant is instructed to rise from the chair, walk three meters at a comfortable and safe pace to a marked line on the floor, turn around, walk back to the chair, and sit down again. The stopwatch starts on the command “Go” and stops when the participant is seated back in the chair. The only equipment needed is a standard armchair and a stopwatch. The time taken to complete the task is noted. Typically, shorter times indicate better mobility. However, specific cut-off times have been suggested for identifying individuals at higher risk of falls: less than 10 s: freely mobile; 10−20 s: mostly independent; 20−29 s: variable mobility; 30 s or more: immobile or at high risk for falls.

### 2.10. tDCS Protocol

The electrical stimulation by the direct current was delivered during the intervention sessions, ten tDCS sessions over two consecutive weeks, with five sessions each week, always at the exact times of the day. Transcranial stimulation was applied using an NKL Microestim tDCS device through two surface sponge electrodes (non-metallic) of 5 × 7 cm^2^, moistened in saline solution. The groups received the intervention as follows:

- Sham transcranial stimulation + exercise protocol
- Anodic stimulation in the Supplementary Motor Area + exercise protocol

**Figure 1:**
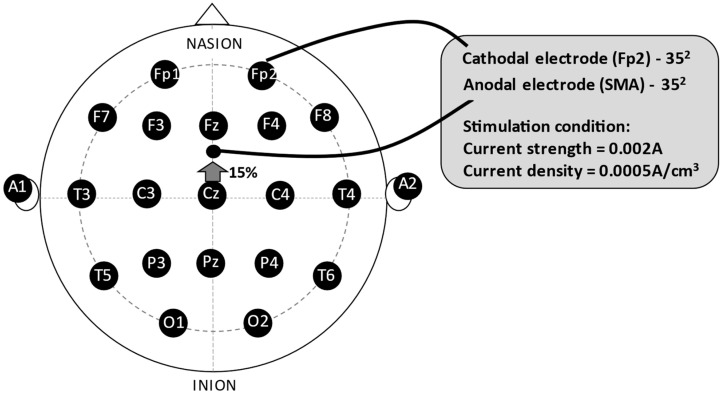
The experimental montage. Legend: SMA = Supplementary Motor Area.

The anode electrode was positioned in front of Cz, at 15% of the nasion-inion distance from the naso-inion measurement at point Cz on the midline, following the international 10−20 system of electroencephalogram, corresponding to the Supplementary Motor Area, and the cathode electrode in the Fp2 region, right supraorbital (Figure 1). All electrode placement procedures were performed in the sham stimulation, but the stimulator was turned on for only 30 s. Hence, the participants experienced the initial sensation of the procedure, but did not receive any stimulation for the remaining time. This procedure is a valid form of control in transcranial direct current-stimulation studies [42,43]. A current of 2 mA (current strength = 0.002 A; current density = 0.0005 A/cm^3^) was delivered over the Supplementary Motor Area for twenty minutes while the participant underwent the exercise protocol. The ramp-up to reach the stipulated intensity of 2 mA was 30 s, as was the ramp-down.

The decision to apply a transcranial direct current stimulation (tDCS) over the Supplementary Motor Area (SMA) was guided by previous studies showing that dysfunction of the SMA has been linked to impaired motor performance in PD, especially in tasks involving self-initiated movements and sequential motor control [44,45]. Additionally, previous studies have demonstrated that stimulating the SMA with tDCS can likely improve motor outcomes, such as movement speed and coordination in PD patients, by enhancing motor preparation and execution [46,47].

Regarding the tDCS montage, the anode was placed over the SMA (15% or approximately 2 cm anterior to Cz, midway between Cz and Fz), and the cathode was positioned over the contralateral supraorbital area, as described in prior studies [46,48]. This montage has been shown to effectively modulate SMA activity with minimal interference from adjacent brain regions. Although the proximity of the electrodes raises concerns about current spread, the size and positioning of the electrodes were selected to focus the current on the SMA while limiting spread [46].

### 2.11. Exercise Protocol

The exercise protocol was based on the Agility Boot Camp (ABC) program developed by researchers at Oregon Health and Sciences University, USA. This program emphasizes training skills such as walking speed, balance, and the relationship between mobility and cognition. Previous research has demonstrated favorable outcomes in variables like mobility, gait speed, balance, and stride length [49]. Although the original studies that described the exercise protocol referred to it as high intensity, they did not define or report how intensity was measured. In our case, we instructed participants to perform the exercises at a self-perceived moderate intensity. However, no objective measures of exercise intensity were recorded. The regimen consisted of five weekly sessions, each lasting 20 min, spanning two consecutive weeks. The participants were familiarized with all exercises before the study, and the chosen tasks aimed to challenge the subjects’ motor and cognitive capacities.

The training regimen comprised walking exercises emphasizing wide steps, arm swings, pelvic dissociation, and direction changes. Upper-limb exercises focused on extension and abduction, emphasizing range of motion. Lower-limb exercises involved maneuvers such as lunges. Obstacle circuits were also integrated into the program, where participants had to surpass or navigate the obstacles with visual cues placed on the ground. Orthostatic exercises were also incorporated, accompanied by an associated trunk rotation and a simulated rowing exercise using a rod.

**Figure 2:**
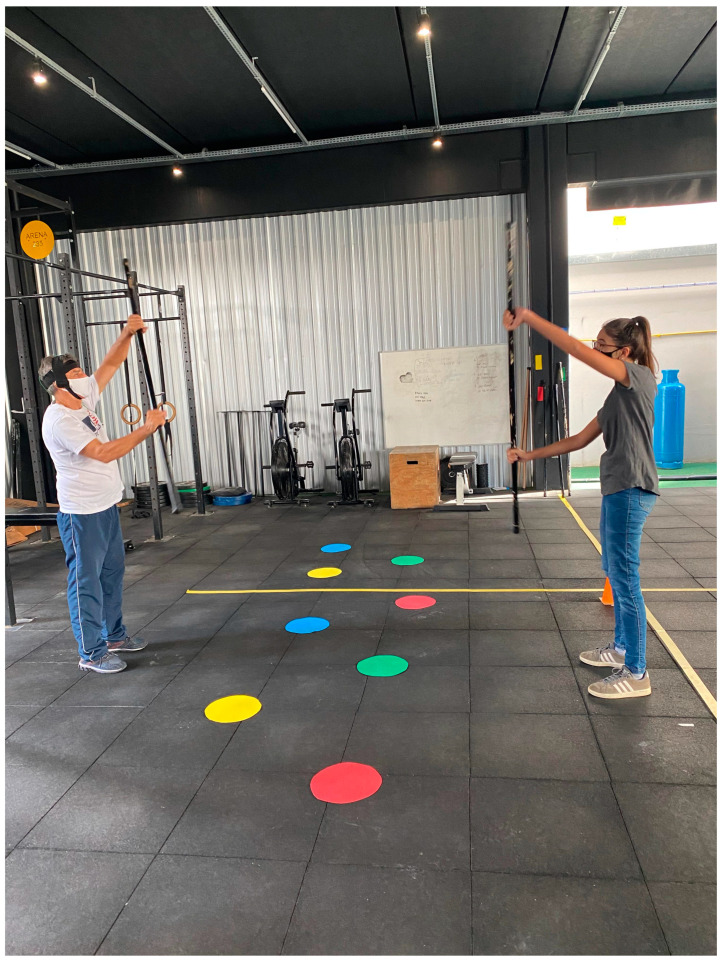
A participant engages in a simulated rowing exercise using a PVC pipe, guided by a therapist. Colored dots on the floor are utilized for dual-task cognitive exercises.

Dual-task cognitive exercises were implemented. Participants walked while performing tasks such as repeating words, naming fruits or people, counting three-digit numbers both forward and backward, or answering the therapist’s questions about colors placed on the visual cues on the floor (Figure 2). All exercises were synchronized with the tDCS application.

A physiotherapist unaware of the study’s objectives recorded participants’ attendance for each session and vital signs before and after the training on a tracking sheet. In every session, participants were asked to complete a questionnaire about possible adverse effects of the previous day’s electrostimulation session. Any issues or complications that arose during the intervention were also documented. Notably, the participants reported no adverse effects throughout the study duration. All participants were advised to maintain their daily activities as usual.

### 2.12. Statistical Analysis

A two-way mixed Analysis of Variance (ANOVA) with repeated measures with one within-subject (time instances: pre and post) and one between-subject factor (groups: intervention, sham, and control) identified the possible main effects and interactions for force and acceleration measures, TUG, and AP displacement. Acceleration’s ApEn, MVC, SD of force, CV of force, RMSE, TUG, and Ap displacement data were log-transformed. Shapiro–Wilk tests were used to test the normality of the model’s residuals, and Levene’s tests were used to verify the homogeneity of variances. Interaction effects between time instances and groups were extracted from the models. Post hoc pairwise comparisons were conducted using estimated marginal means with the “emmeans” package in R. Bonferroni adjustments were used to adjust for multiple comparisons.

We first used a three-way ANOVA with repeated measures for the force and acceleration coherence, adding a within-subject factor to account for the four frequency bands investigated. However, even after transforming the data, we could not pass the normality of the residual task, so we ran a linear mixed-effects model (LMM) with Markov Chain Monte Carlo (MCMC) sampling to account for the non-normality of residuals. The LMM included three fixed effects: time instances (pre, post), groups (intervention, sham, control), and frequency bands (0–1, 1–4, 4–8, and 8–12 Hz) along with all possible two- and three-way interactions. The random intercepts for each subject were included to account for repeated measures within individuals. MCMC sampling was performed with 10,000 iterations and two chains to obtain the posterior distributions of the fixed effects. Convergence was assessed using potential scale reduction factors (PSRF), and credible intervals for the fixed effects were computed using the Highest Posterior Density (HPD) intervals. Significant differences were identified based on MCMC-derived *p*-values and the 95% credible intervals that did not contain zero.

Correlation analyses were employed to explore potential relationships between crucial outcome variables, such as improvements in mobility (measured by TUG) and tremor–force decoupling. Pearson’s correlation coefficient (or Spearman’s rank correlation, where applicable) was used to quantify the strength and direction of associations between these variables across participants.

All statistical tests were set with an alpha level of 0.05. Data are presented as mean ± standard error in the text and figures. Unless otherwise stated, only the significant main effects and interactions are provided. The statistical software IBM SPSS (version 20.0, IBM Corp., Chicago, IL, USA) and Visual Studio Code (v. 1.92.1) using R (v. 4.4.1) were employed for these analyses.

## 3. Results

### 3.1. Participant Characteristics

Table 2 presents the anthropometric characteristics of participants and their classification according to the Hoehn and Yahr (H&Y) scale.

### 3.2. Motor Control and Acceleration Profile

Motor control and acceleration profiles did not show significant differences. The global mean results are presented in Table 3.

### 3.3. Functional Limit Test

**Figure 3:**
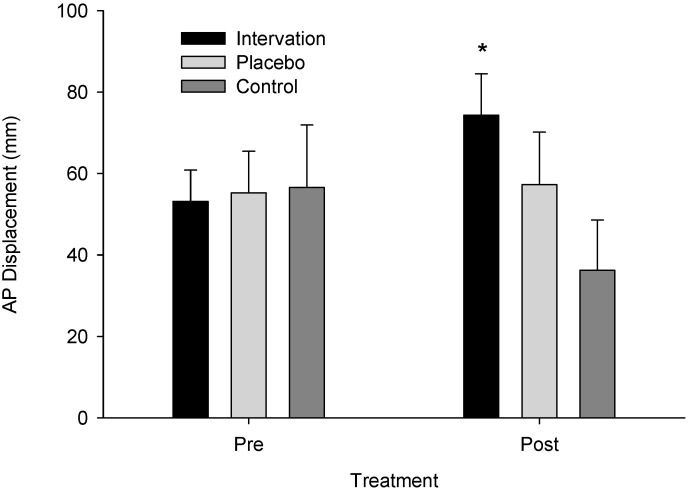
Anterior–posterior displacement across groups pre- and post-treatment. An asterisk (*) indicates a significant difference from pre- to post-treatment for the intervention group (*p* = 0.027).

Analysis of variance model assumptions for the log-transformed AP Displacement data were verified (Shapiro–Wilk (W)= 0.985, *p* = 0.775; Levene’s F(5,44) = 1.421, *p* = 0.235) and a significant time instances–x-group interaction was found (F(2,22) = 4.774, *p* = 0.019, η_p_^2^ = 0.303, observed power = 0.735). The post hoc analysis indicated that the intervention group had higher post-treatment than pre-treatment AP Displacement values (*p* = 0.027) (Figure 3). Interestingly, the control group exhibited lower AP displacement values in the second measurement than the first (*p* = 0.025). Mean anterior–posterior displacement pre-intervention was 53.000 (SE = 11.748, CI: 28.64–77.36) and post-intervention was 74.375 (SE = 11.875, CI: 49.747–99.003) for the intervention group, 55.375 (SE = 11.748, CI: 31.01–79.739) and 57.375 (SE = 11.875, CI: 32.747–82.003) for the sham group, and 56.509 (SE = 11.076, CI: 33.538–79.479) and 36.343 (SE = 11.196, CI: 13.124–59.563) for the control group, respectively.

### 3.4. Time up and Go Test

**Figure 4:**
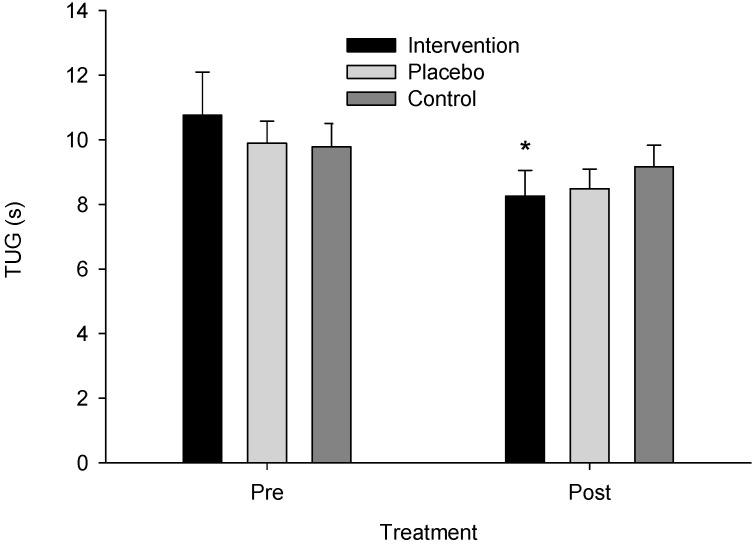
Time Up and Go test (TUG) scores across groups pre- and post-treatment. An asterisk (*) indicates a significant difference from pre- to post-treatment for the intervention group (*p* < 0.001).

Similarly, ANOVA model assumptions for the log-transformed TUG data were verified (W = 0.974, *p* = 0.331; Levene’s F(5,44) = 0.810, *p* = 0.549), and a significant time instances–x-group interaction was found (F(2,22) = 5.447, *p* = 0.012, η_p_^2^ = 0.331, observed power = 0.793). The post hoc analysis indicated that the intervention group had lower post-treatment than pre-treatment TUG values (*p* < 0.001) (Figure 4). Mean TUG pre-intervention was 10.756 (SE = 0.954, CI: 8.778–12.735) and post-intervention was 8.258 (SE = 0.690, CI: 6.827–9.688) for the intervention group, 9.891 (SE = 0.954, CI: 7.913–11.870) and 8.486 (SE = 0.690, CI: 7.056–9.917) for the sham group, and 9.778 (SE = 0.899, CI: 7.912–11.643) and 9.167 (SE = 0.650, CI: 7.818–10.515) for the control group, respectively.

### 3.5. Acceleration × Force Wavelet Coherence

**Figure 5:**
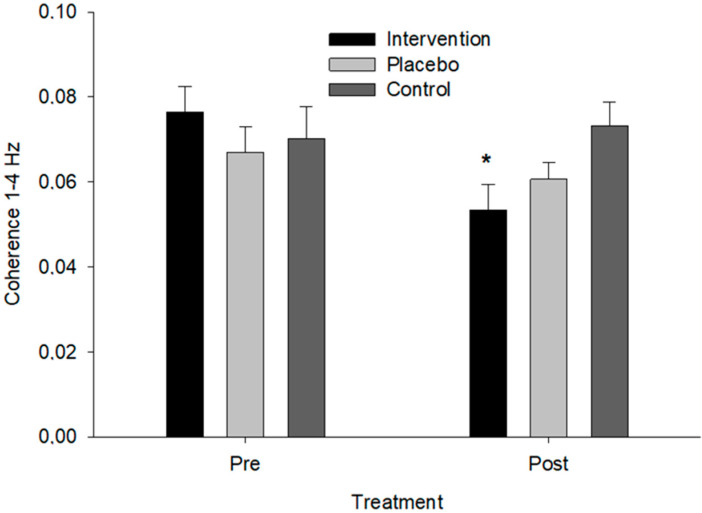
Coherence between force and acceleration in the 1–4 Hz frequency band across groups. An asterisk (*) indicates a significant difference from pre- to post-treatment for the intervention group (*p* = 0.007).

For the Acc × force wavelet coherence analysis, the LMM revealed a significant three-way interaction between time instances, groups, and frequency bands (MCMC *p* = 0.007). The specific interaction between intervention, post-timepoint, and the 1–4 Hz frequency band indicated a significant decrease in coherence in this frequency band for the Intervention group from pre to post compared to the sham and control groups. No significant changes were observed in other frequency bands (*p* > 0.05) or between the sham and control groups (*p* > 0.05). The posterior distributions for the fixed effects supported these findings, as the 95% credible intervals for the significant interaction did not include zero, confirming the robustness of the observed effects. The potential scale reduction factors (PSRF) were close to 1, indicating good convergence of the MCMC chains. (Figure 5). The estimated marginal mean for the pre-intervention acceleration force coherence at 1–4 Hz was 0.071 (SE = 0.005, CI: 0.060–0.082) and post-intervention was 0.059 (SE = 0.005, CI: 0.049–0.070) for the intervention group, 0.069 (SE = 0.005, CI: 0.058–0.080) and 0.065 (SE = 0.005, CI: 0.054–0.076) for the sham group, and 0.073 (SE = 0.005, CI: 0.062–0.083) and 0.072 (SE = 0.005, CI: 0.061–0.082) for the control group, respectively.

### 3.6. Correlation Analysis

**Figure 6:**
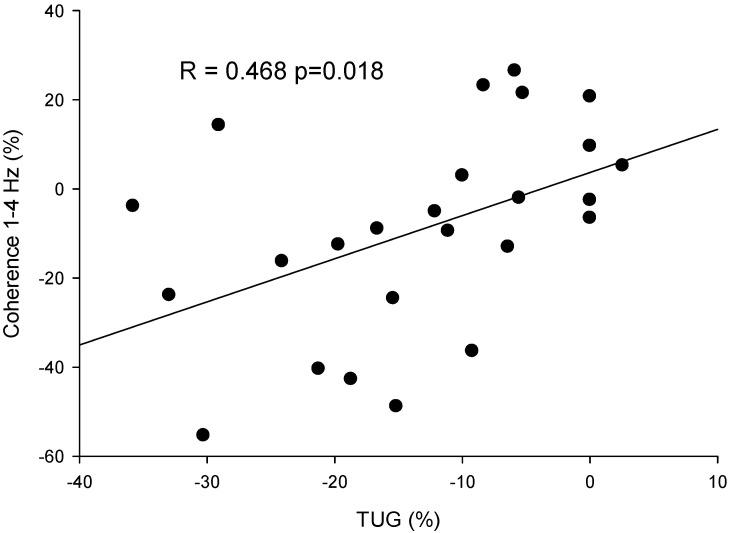
Scatter plot of the percentage variation pre–post-intervention in the coherence between force and acceleration in 1–4 Hz and the score in the Time Up and Go test (TUG) for all participants (dots).

The correlation analyses indicated that the changes in TUG from post- to pre-treatment were significantly positively correlated with the changes in coherence (R = 0.468, *p* = 0.018), indicating that increased mobility was associated with the decoupling of tremor and force control (Figure 6).

## 4. Discussion

This study aimed to determine how combining tDCS with exercise affects PD patients’ functional mobility and tremor control. The primary findings demonstrated that this combination significantly improved balance stability, as reflected by increased anterior–posterior (AP) displacement, mobility reflected by TUG performance, and tremor-force decoupling compared to the sham and control groups over two weeks. Notably, the analysis also revealed a significant positive correlation between changes in force–tremor decoupling and TUG performance. One main limitation of the study was the absence of a group receiving only tDCS.

In recent years, non-invasive brain stimulation (NIBS) techniques have gained significant attention for their ability to modulate cortical excitability across various clinical and experimental settings. These techniques are valuable for enhancing motor functions [50,51,52,53] and investigating behavioral causality [54]. Anodal transcranial direct current stimulation (tDCS), a type of NIBS, administers a steady, facilitatory electrical current to the brain via an anodal electrode placed over the target cortical region, being shown to be capable of enhancing various motor functions in both healthy individuals [51,55,56,57] and patients with PD [13,58].

The SMA is recognized as a crucial cortical region involved in the preparation and initiation of self-initiated movements [44,59] and the coordination of movement and posture during motor tasks [50,60,61]. In PD patients, reduced SMA activity has been observed due to decreased positive efferent feedback from the basal ganglia–thalamocortical motor loop [45,62,63], potentially leading to impaired motor functions [63]. The current study has shown promising functional outcomes from combining anodal tDCS with the SMA and exercise. Notwithstanding, our exercise protocol involved therapeutic exercises and dual-task activities, which is essential considering the increased SMA activity produces an additive effect, enhancing motor performance more significantly when combined with therapeutic exercise than exercise alone. These findings align with previous research demonstrating that NIBS of the SMA alleviates PD-related symptoms [64] and improves motor functions [47,65].

Furthermore, the present study demonstrates that increased cortical excitability, when integrated with specific training programs, significantly enhances the effectiveness of motor practice, resulting in improved functional outcomes compared to practice alone. Given the established link between increased cortical excitability and improvements in functional mobility in PD patients [42,46], the group receiving tDCS may have benefited more from each training session, accumulating a more significant overall effect over two weeks than the sham and control groups. In particular, anodal tDCS applied to the SMA may transiently elevate SMA excitability, optimizing practice effects and enhancing outcomes at each session. The tDCS and exercise group demonstrated a faster rate of functional improvement than the other groups.

Alternatively, given the strong connections between the SMA and basal ganglia, tDCS-induced dopamine release may account for the enhanced rehabilitative outcomes observed in this study [45,62,63], particularly when combined with exercise. This aligns with prior research demonstrating that dopamine replacement therapy and deep brain stimulation can effectively restore reduced SMA activity in PD patients [62,66,67], underscoring the reciprocal relationship between the SMA and basal ganglia [68]. In contrast to various findings in PD studies where NIBS was applied to the SMA, the present study demonstrated promising outcomes when tDCS was combined with exercise, compared to exercise alone. However, the absence of a control group receiving only tDCS represents a limitation in determining the isolated effects of tDCS applied to the SMA in improving functional outcomes in PD patients. Future studies are essential to assess the efficacy of multiple sessions of tDCS targeting the SMA in enhancing functional outcomes among PD patients, taking into account key factors such as the timing, duration, and intensity of tDCS administered during each training session. These insights will help optimize therapeutic and rehabilitative strategies for PD, potentially improving functional outcomes of PD patients by refining non-invasive neuromodulation techniques for motor function recovery.

Interestingly, the current results further demonstrate a positive correlation between changes in TUG task performance and the extent of force–tremor coupling. Specifically, increased bilateral decoupling between force and tremor is associated with decreased time to complete the TUG task. Given that the SMA plays a vital role in mediating interhemispheric interactions [69], the increased decoupling between force produced in one limb and tremor produced in the opposite limb may result from enhanced interhemispheric inhibition, weakening the adverse influence of tremor on the force production of the contralateral limb. One previous study has also demonstrated that the modulation of the SMA results in the change in interhemispheric communication between the bilateral motor cortex [46]. The increased inhibition of the contralateral influence could result from the enhancement of the SMA activity, facilitating motor performance. Considering the role of the SMA in influencing functional mobility, the enhanced SMA function could also lead to a decreased time to complete the TUG task, supported by the previous study [46]. Thus, the correlation between changes in force–tremor decoupling and improvement in the TUG task can reflect enhanced SMA excitability, underscoring the multifaceted role of the SMA in motor control and functional mobility. Thus, the observed correlation between changes in force–tremor decoupling and improved TUG task performance can be attributed to the latent effect of increased SMA excitability, highlighting its multifaceted role in motor control and functional mobility.

The lack of improvement in the exercise-only group after ten days is likely due to the relatively short duration of the intervention. Previous studies suggest that exercise interventions in PD often require longer durations to yield significant improvements in motor and cognitive function. For instance, studies have shown that benefits from exercise may become apparent after several weeks of consistent training rather than within a 10-day window [70,71]. A meta-analysis by Meserve et al. [72] also found that improvements in gait and balance typically require interventions of at least four to six weeks. Therefore, the short intervention period in this study may have been insufficient to detect measurable improvements in the exercise-only group.

Despite the significant improvements in functional outcomes induced by combining tDCS and therapeutic exercise, this investigation remains constrained by several limitations. As noted earlier, the isolated efficacy of tDCS applied to the SMA in improving functional outcomes remains unclear. The absence of a control group receiving only tDCS limits the ability to disentangle the specific contributions of tDCS and exercise in improving functional outcomes. Furthermore, the current findings do not fully address the underlying neural mechanisms driving the effectiveness of the combined tDCS and exercise intervention. It is possible that tDCS targeting the SMA, in conjunction with exercise, influences training outcomes through two mechanisms: (1) at each session, transient increases in SMA excitability, induced by anodal tDCS, directly enhance motor performance, resulting in a more substantial cumulative effect over the two-week training period; (2) restoration of SMA excitability may occur through remote activation of dopaminergic neurons, indirectly modulating the connectivity of SMA-BG pathways [45,49,62,68]. Following multiple sessions of tDCS, resting SMA excitability may be significantly enhanced compared to pre-intervention levels, leading to improved functional outcomes. Future studies should aim to elucidate these mechanisms in greater detail. One limitation of our study is the need for more direct measurement and control of exercise intensity across participants and conditions. This approach is consistent with previous studies in the field, which also did not measure exercise intensity objectively [49,73]. Still, we acknowledge that this may introduce variability in participant exertion levels. Future studies should consider incorporating more objective methods, such as heart rate monitoring or accelerometry, to control and measure exercise intensity more accurately. Finally, the present study’s relatively small sample size may have resulted in insufficient statistical power to detect more minor effects, particularly in subgroup analyses. As we aimed for a medium effect size based on the previous literature and considering recruitment challenges, non-significant findings should be interpreted cautiously, and further studies with larger samples are recommended to confirm these results.

## 5. Conclusions

In conclusion, combining anodal tDCS with the SMA and therapeutic exercise improved balance stability, functional mobility, and force–tremor decoupling. No changes were observed in the exercise-alone or control groups. These data further support the crucial role of the SMA in motor functions in PD and demonstrate the efficacy of combining therapeutic training with the NIBS approach in rehabilitative settings. Future research must explore the underlying neural mechanisms induced by such combined interventions that contribute to behavioral changes.

## Figures and Tables

**Table 1:** Patients’ medication and the LEDD for all participants.

Group	Prolopa 200 mg + Benserazide 50 mg (Pills per Day)	Prolopa 100 mg + Benserazide 50 mg (Pills per Day)	Prolopa Hbs (Pills per Day)	Entacapona 200 mg (Pills per Day)	Pramipexol 0.25 mg (Pills per Day)	LEDD
Control	2	0	0	0	0	400
Control	0	4	0	0	0	400
Control	2	0	0	0	0	400
Control	2.5	0	0	0	0	500
Control	2	0	0	0	0	400
Control	3	0	0	0	0	600
Control	2	0	0	0	0	400
Control	2	0	0	0	0	400
Control	2	0	0	0	0	400
Intervention	2	0	0	0	0	400
Intervention	2	0	0	0	0	400
Intervention	2	0	0	0	0	400
Intervention	4	0	0	0	0	800
Intervention	4	0	0	0	3	1200
Intervention	0.5	0	0	0	0	100
Intervention	3	0	0	3	0	1200
Intervention	4	0	1	0	0	800
Sham	3	0	0	0	0	1400
Sham	3	0	0	0	0	600
Sham	2	0	0	0	0	400
Sham	2	0	0	0	0	400
Sham	8	0	0	0	0	1600
Sham	2	0	0	0	0	400
Sham	0	4	0	0	0	400
Sham	2	0	0	0	0	400

Legend: LEDD: Levodopa Equivalent Daily Dose.

**Table 2:** Anthropometric characteristics of participants and their classification according to the Hoehn and Yahr (H&Y) scale.

Characteristics	Intervention Group (Mean ± SD)	Sham Group (Mean ± SD)	Control Group (Mean ± SD)	*p*-Value
Age (years)	69.62 ± 0.76	64 ± 2	65.12 ± 9.7	0.578 #
Weight (kg)	72.87 ± 12.73	62.2. ± 17.19	72.75 ± 7.8	0.412 #
Height (cm)	165.25 ± 7.75	165.8 ± 7.49	169.1 ± 6.72	0.356 #
Sex (M:F)	6:2	4:4	5:4	
H&Y score	2.75 ± 0.46	2.6 ± 0.54	2.22 ± 0.44	0.093 @

Legend: SD = standard deviation; kg = kilograms; cm = centimeter; M = male; F = female. # One-way Analysis of Variance; @ Median Test for k samples.

**Table 3:** Mean and standard deviation results for motor control and acceleration profile pre- and post-intervention.

Measure	Intervention	Sham	Control	*p*/η_p_^2^
Dominant Freq AccR	Pre: 6.52 ± 0.58	6.51 ± 0.68	6.98 ± 0.52	0.965/0.003
	Post: 6.30 ± 0.69	6.41 ± 0.78	6.83 ± 0.73	
ApEn AccR_f	1.36 ± 0.37	1.19 ± 0.46	0.82 ± 0.15	0.527/0.057
	1.30 ± 0.49	1.25 ± 0.44	0.87 ± 0.18	
MVC (N)	Pre: 178.5 ± 94.4	137.3 ± 65.9	139.7 ± 47.3	0.230/0.125
	Post: 195.6 ± 100.3	134.8 ± 50.9	118.3 ± 42.18	
Mean Force (N)	Pre: 35.33 ± 18.39	23.50 ± 11.99	26.69 ± 6.82	0.061/0.224
	Post: 39.49 ± 20.94	25.63 ± 8.90	21.80 ± 8.02	
SD Force (N)	Pre: 0.96 ± 0.58	1.42 ± 2.07	1.36 ± 1.28	0.397/0.080
	Post: 1.25 ± 0.75	0.90 ± 0.51	1.07 ± 1.12	
CV Force	Pre: 2.83 ± 1.26	5.35 ± 5.82	5.10 ± 5.04	0.569/0.050
	Post: 3.60 ± 2.20	3.93 ± 3.24	4.83 ± 4.43	
RMSE Force (N)	Pre: 1.32 ± 0.44	1.83 ± 2.16	1.96 ± 0.97	0.082/0.203
	Post: 1.98 ± 1.33	1.26 ± 0.46	1.01 ± 0.74	

Legend: Freq: frequency; AccR: acceleration in resultant direction; ApEn AccR_f: approximate entropy of filtered resultant acceleration; MVC: maximum voluntary contraction; SD: standard deviation; CV: coefficient of variation; RMSE: root mean square error; *p*: *p*-value from intervention × time interaction, η_p_^2^: partial eta squared (effective size) from intervention × time interaction.

## Data Availability

Data used in this work are available under request to the corresponding author.
